# Mechanical, physico-chemical, and antimicrobial properties of gelatin-based film incorporated with catechin-lysozyme

**DOI:** 10.1186/1752-153X-6-131

**Published:** 2012-11-07

**Authors:** Saroat Rawdkuen, Phunsiri Suthiluk, Damrongpol Kamhangwong, Soottawat Benjakul

**Affiliations:** 1 Food Technology Program, School of Agro-Industry, Mae Fah Luang University, Muang, Chiang Rai, 57100, Thailand; 2 Technology Management of Agricultural Produces and Packaging Program, School of Agro-Industry, Mae Fah Luang University, Muang, Chiang Rai, 57100, Thailand; 3 Department of Food Technology, Faculty of Agro-Industry, Prince of Songkla University, Hat Yai, Songkhla, 90112, Thailand

**Keywords:** Antimicrobial, Biodegradable packaging, Catechin, Gelatin film, Lysozyme

## Abstract

**Background:**

Microbial activity is a primary cause of deterioration in many foods and is often responsible for reduced quality and safety. Food-borne illnesses associated with *E*. *coli* O157:H7, *S*. *aureus*, *S*. *enteritidis* and *L*. *monocytogenes* are a major public health concern throughout the world. A number of methods have been employed to control or prevent the growth of these microorganisms in food. Antimicrobial packaging is one of the most promising active packaging systems for effectively retarding the growth of food spoilage and pathogenic microorganisms. The aim of this study was to determine the mechanical, physico-chemical properties and inhibitory effects of the fish gelatin films against selected food spoilage microorganisms when incorporated with catechin-lysozyme.

**Results:**

The effect of the catechin-lysozyme combination addition (CLC: 0, 0.125, 0.25, and 0.5%, w/v) on fish gelatin film properties was monitored. At the level of 0.5% addition, the CLC showed the greatest elongation at break (EAB) at 143.17% with 0.039 mm thickness, and the lowest water vapor permeability (WVP) at 6.5 x 10^−8^ g·mm·h^-1^·cm^-2^·Pa^-1^, whereas the control showed high tensile strength (TS) and the highest WVP. Regarding color attributes, the gelatin film without CLC addition gave the highest lightness (L* 91.95) but lowest in redness (a*-1.29) and yellowness (b* 2.25) values. The light transmission of the film did not significantly decrease and nor did film transparency (p>0.05) with increased CLC. Incorporating CLC could not affect the film microstructure. The solubility of the gelatin based film incorporated with CLC was not affected, especially at a high level of addition (p>0.05). Inhibitory activity of the fish gelatin film against *E*.*coli*, *S*.*aureus*, *L*. *innocua* and *S*. *cerevisiae* was concentration dependent.

**Conclusions:**

These findings suggested that CLC incorporation can improve mechanical, physico-chemical, and antimicrobial properties of the resulting films, thus allowing the films to become more applicable in active food packaging.

## Background

Microbial activity is a primary cause of deterioration in many foods and is often responsible for reduced quality and safety. Food-borne illnesses associated with *E*. *coli* O157:H7, *S*. *aureus*, *S*. *enteritidis* and *L*. *monocytogenes* are a major public health concern throughout the world [1]. A number of methods have been employed to control or prevent the growth of these microorganisms in food, including the use of physical, biological, or chemical procedures. However, due to increasing concerns over the use of chemical agents as well as the high cost of physical treatments, there has been an increased demand for naturally-derived antimicrobial substances for food industries. Packaging incorporated with naturally active compounds is an alternative way to prolong shelf life of any processed foods. In general, packaging is needed for the protection of food from the outside environment and/or from post contamination by microorganisms [2]. Recently, much attention has been paid to the development of antimicrobial active systems by means of incorporating antimicrobial substances into and on food or onto the coatings of food packaging, which help to improve food safety and shelf life [3]. Antimicrobial packaging is one of the most promising active packaging systems for effectively killing, inhibiting, or retarding the growth of food spoilage and pathogenic microorganisms. By these actions, the shelf life of the product is prolonged, and its quality and safety are better preserved [4].

Naturally occurring antimicrobials have been widely incorporated in gelatin film, including chitosan [5,6], lysozyme [7], essential oils [8,9], and nisin [10]. Green tea extract has also been used as an active ingredient for active film preparation because of the polyphenols in tea that are good for both their antioxidant and antimicrobial activities [11,12]. The active compounds can inhibit microorganism growth and food spoilage and thereby extend shelf life of processed foods by either reducing the microbial growth rate or extending the lag-phase of the target microorganisms [12,13]. However, addition of these compounds to the film based material may affect the principal properties of the host material. Many researchers have reported that various bioactive additives such as enzymes [7], antimicrobial [5,6], and antioxidant agents [11,12] may get entrapped in the porous solid matrices in the gelatin and also be immobilized by covalent binding to a solid support, which would result in a change to the resulting film properties [14,15].

Among all materials that are used for biodegradable film preparation, proteins are considered to provide the most desirable mechanics, gas barrier, and transparency properties, as well as provide high nutritional value [16]. Gelatin-based films are thin, flexible, and transparent biodegradable materials. There are many applications for this type of film in food processing, packaging, drug recovery, and more [4,8,9,17,18]. The physical and structural properties of gelatin film are mainly influenced by a variety of factors such as molecular weight distribution, amino acid composition, plasticizer, and additives used, which play an important role in the mechanical, optical, and barrier properties of the resulting films [19]. The addition of some other active compounds can alter the physico-chemical properties of the film, such as increased flexibility and moisture sensitivity, as well as other functional properties. The purpose of this study was to determine the mechanical, physico-chemical properties and inhibitory effects of the fish gelatin films against selected food spoilage microorganisms when incorporated with catechin-lysozyme.

## Results and discussion

### Effect of CLC on mechanical and physical properties of film

#### Tensile strength and elongation at break

TS and EAB of gelatin films incorporated with CLC at various concentrations are shown in Table 1. The TS of the resulting films were affected by the addition of CLC. The presence of CLC in film structure caused the marked decrease in TS, especially at the level of 0.5% incorporation. At the same time, when this concentration was incorporated into the film, a huge amount of EAB was increased by about 5 times compared to the control film. This was attributed to the interaction between CLC and the gelatin molecules, which resulted in a modification to the protein network in the film. No significant difference of EAB was observed between the control film and the film added with CLC at the levels of 0.125 to 0.25% (p>0.05). Ahmad et al. [9] suggested that the incorporation of other polymeric components at an excessive amount is more likely a result of the development of a heterogeneous film structure, which feature discontinuities or irregularities. Nevertheless, the additive compound at an appropriate level could strengthen the film matrix by enhancing the interaction between protein chains. Film formation generally takes place by the development of a three dimensional network of protein molecules: ionic, hydrophobic, hydrogen and disulfide bonds [20]. Cagri et al. [21] reported that incorporation of additives other than cross-linking agents generally lowers TS. In the present study, TS and EAB were decreased with increasing CLC levels not over 0.5% and 0.25%, respectively. It is suggested that the CLC contained different compounds, especially in lysozyme, thereby affecting protein interaction differently. From this result, high levels of CLC incorporation may help to improve the plasticizing effect of the gelatin film, consequently enhancing the film’s stretch ability.

**Table 1:** Mechanical properties of fish gelatin based films incorporated with a combination of catechin and lysozyme at different concentrations

**Treatment (% CLC)**	**Tensile strength (MPa)**	**Elongation (%)**
Control	33.49 ± 2.63 ^c^	27.82 ± 10.52 ^a^
0.125 %	34.34 ± 4.80 ^c^	19.90 ± 9.40 ^a^
0.250 %	26.95 ± 4.26 ^b^	19.21 ± 11.84 ^a^
0.500 %	3.31 ± 0.45 ^a^	143.17 ± 7.81 ^b^

Values (*n* = 10) with different superscripts in each column are significantly different (p<0.05).

#### Thickness

The thickness of gelatin films incorporated with CLC at various concentrations is shown in Table 2. There showed no significant increase (0.125-0.5%) when compared with the control (p>0.05). It ranged from 0.036 to 0.039 mm. The results suggested that CLC could not form the compact film network with gelatin molecules, which resulted in no difference in film thickness. A protruded structure that formed during the film formation might have been induced by the compounds in the CLC, which could have interacted with the gelatin to form agglomerates or particulates. According to Han and Krochta [22], film thickness is influenced by the solid content of the film forming solution. Film thickness generally affects film properties such as mechanical properties, water vapor permeability, light transmission, and film transparency.

**Table 2:** Thickness and water vapor permeability of fish gelatin based films incorporated with a combination of catechin and lysozyme at different concentrations

**Treatment** (% **CLC**)	**Thickness** (**mm**)	**WVP (*n=*5)(10**^−**8**^**g**·**mm**·**h**^-**1**^·**cm**^-**2**^·**Pa**^-**1**^)
Control	0.036 ± 0.0012 ^a^	12.60 ± 0.33 ^d^
0.125 %	0.038 ± 0.0021 ^b^	10.77 ± 0.71 ^c^
0.250 %	0.037 ± 0.0022 ^ab^	8.43 ± 0.53 ^b^
0.500 %	0.039 ± 0.0022 ^b^	6.50 ± 0.43 ^a^

Values (*n* = 10) with different superscripts in each column are significantly different (p<0.05).

### Effect of CLC on physico-chemical properties of film

#### Water vapor permeability

The WVP of the gelatin based film incorporated with CLC at various concentrations is presented in Table 2. The differences in WVP were observed among films prepared from different CLC concentrations (p<0.05). When increasing the CLC concentration incorporated into the film, decreased WVP was observed. The WVP of the resulting films decreased from 12.60 (control) to 6.50 (0.5% CLC), which shows the highest and lowest WVP of the films, respectively. The structural modifications of the gelatin network might be occurred when CLC was added. The lysozyme peptide or functional group of catechin will react chemically with a gelatin to form a stable bond, which results in the formation of a more complex structure of the resulting film. A high WVP of edible film is not desirable with respect to its usage and performance [5]. From the results, the addition of CLC could improve the barrier properties of the film by lowering moisture transfer between the food and the surrounding atmosphere when this film was applied to any heterogeneous food product. The water vapour transfer process in films depends on the hydrophilic-hydrophobic ratio of the film constituents [9]. Additionally, more pronounced protein cross-linking of the film along with more rigid and denser structures might retard water diffusion through the films. Film with higher thickness can absorb more water from the environment [23]. These results are in contrast with the results of Bower et al. [7], who reported that when the concentration of lysozyme concentration increased from 0 to 0.1%, decreased of WVP was observed in antimicrobial gelatin film.

#### Color

Color attributes are of prime importance because they directly influence consumer acceptability. The lightness (L*), redness/greenness (a*) and yellowness/blueness (b*) values of gelatin based films incorporated with different concentrations of CLC are presented in Table 3. The color parameter of the films was affected by the levels of CLC incorporation. Increased lightness values (88.43 to 88.98) coincidentally decreases in redness values (−0.01 to −0.41) in films incorporated with 0.125-0.5% CLC. However, when compared with the control film, it showed a higher value than that of the film incorporated with CLC. In addition, a marked increased of yellowness values (control: 2.25, 0.5% CLC: 5.44) was observed when the concentration of CLC increased (p<0.05). The results demonstrated that the color parameters of the resulting films were affected by CLC addition. The increase in lightness values in the film incorporated with CLC was probably due to the light scattering effect of the matrix compound of CLC and gelatin in the film. In addition, the color formation in the resulting film may have been caused by the chemical alterations in the biomaterials used for film preparation.

**Table 3:** Color parameters of fish gelatin based films incorporated with a combination of catechin and lysozyme at different concentrations

**Treatment (% CLC)**	L^*^	a^*^	b^*^
Control	91.95 ± 0.08 ^c^	−1.29 ± 0.01 ^a^	2.25 ± 0.07 ^a^
0.125 %	88.43 ± 0.43 ^a^	−0.01 ± 0.06 ^d^	3.88 ± 0.34 ^b^
0.250 %	88.62 ± 0.26 ^ab^	−0.15 ± 0.14 ^c^	5.02 ± 0.23 ^c^
0.500 %	88.98 ± 0.51 ^b^	−0.41 ± 0.05 ^b^	5.44 ± 0.11 ^d^

Values (*n* = 5) with different superscripts in each column are significantly different (p<0.05).

#### Light transmission and transparency

Light transmission in UV (200–280 nm) and visible ranges (350–800 nm), as well as the transparency of the film samples incorporated with different concentrations of CLC, are shown in Table 4. Generally, all films exhibited lower light transmission in the UV range than in the visible range. The light transmission in the UV range was from 0.1 to 14.14%, while the transmission in the visible range was from 64.6 to 84% and 37.1 to 87.4% in the control and film incorporated with CLC, respectively. An insignificant increases of light transmission was observed in the film when CLC was incorporated (p>0.05). In addition, increased CLC concentration also had no significant effect to the light transmission of the film. This result suggested that gelatin-based films incorporated with CLC could prevent UV transmission and therefore retard lipid oxidation induced by UV light in a food system. This result was consistent with Jongjareonrak et al. [24] who observed low transmissions of light in the UV range of fish gelatin-based films. Optical properties of films are an important attribute that influences its appearance, marketability, and their suitability for various applications. Clear edible films are typically desirable with higher applicability and acceptability in food packaging systems [9].

**Table 4:** Light transmission and transparency of fish gelatin based films incorporated with a combination of catechin and lysozyme at different concentrations

**Treatment (% CLC)**	**Wavelength (nm)**								**Transparency values**
	**200**	**280**	**350**	**400**	**500**	**600**	**700**	**800**	
Control	0.1	14.4	64.6	75.1	79.8	81.9	83.3	84.0	3.34 ± 0.01 ^ab^
0.125 %	0.1	0.1	41.2	71.1	80.5	84.1	85.9	87.1	3.36 ± 0.01 ^ab^
0.250 %	0.1	0.1	37.1	69	80.5	84.3	86.5	87.4	3.34 ± 0.01 ^ab^
0.500 %	0.1	0.1	40.6	67.6	79.3	82.8	85.1	86.8	3.33 ± 0.01 ^a^

Values are given as mean ± SD from triplicate determinations.

Values with different superscripts in the column are significantly different (p<0.05).

**Figure 1:**
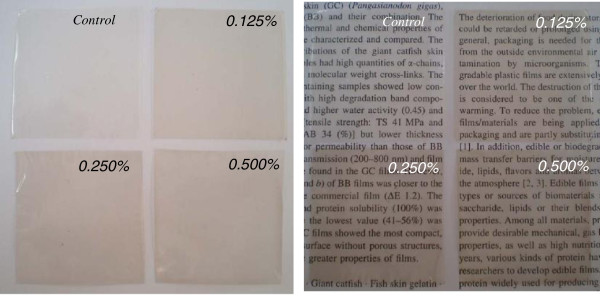
Surface morphology of fish gelatin based film incorporated with a combination of catechin and lysozyme at different concentrations.

For film transparency, no significant differences between treatments and the control were observed (p>0.05) (Table 4). This result was also confirmed by the surface morphology with different backgrounds of the films in Figure 1. The higher transparency value indicated that the film was less transparent. From the result, it was found that incorporating CLC into the gelatin based film did not affect the transparency of the resulting films. This indicated that the transparency properties of the gelatin based films still remained even though CLC was incorporated. Transparency of fish gelatin film with or without the addition of any active compounds ranged from 2.5 to 3.3 [9,23].

#### Surface morphology and film microstructure

The surface morphology of gelatin based film incorporated with CLC at various concentrations is shown in Figure 1. All film specimens were highly transparent, but some of them, although highly translucent, presented a slightly yellow to brown appearance. No significant differences between samples were observed in terms of transparency as indicated by the appearance of letters on the background. However, there was more turbidity of the gelatin film present in the white background when the concentration of CLC increased compared to the control, especially for the film containing 0.5% CLC. These results were similar to the light transmission and transparency of the gelatin film as mentioned above. Based on the film surface morphology property, applications of edible gelatin film incorporated with CLC at high levels may be limited to only some food products e.g. red meat products or sausages. According to the transparency of the film, customers can clearly see the product packed inside the package, making it is easier deciding whether or not to buy that product.

**Figure 2:**
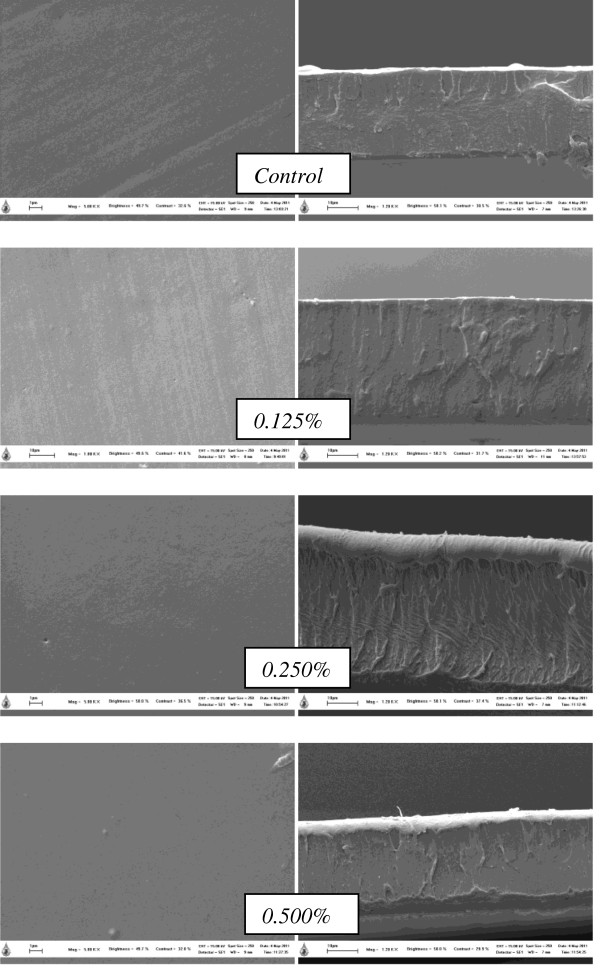
**SEM micrographs of fish gelatin based film incorporated with a combination of catechin and lysozyme at different concentrations.** The magnification is 5,000x for the surface and 1,200x for the cross section films.

SEM micrographs of surface gelatin film incorporated with CLC at various concentrations are shown in Figure 2. The film had a clean, compact, smooth and continuous surface without grainy and porous structures. Increased CLC concentration did not affect the film morphology. The excellent structural integrity of the film was comparable with that of the film from giant catfish skin and bovine bone gelatins [23]. This result indicated that an ordered matrix structure of the film component managed to form. It was also found that no interfaces were observed in the blend or in the bi-layer film, indicating a high compatibility between components, yielding a relatively smooth and compact morphology. From the result, it was hypothesized that interaction between gelatin molecules and CLC could take place at the interface of the bi-layer film. These results suggested that the compact structure of the resulting films might be responsible for the film properties, especially for the WVP, oxygen permeability, and/or other physico-chemical properties of the resulting films.

#### Film solubility and protein solubility

Solubility of gelatin film incorporated with different concentrations of CLC in terms of water and protein solubility is shown in Table 5. The control film showed the highest film solubility (57.51%) and protein solubility (54.78%), while film incorporated with CLC exhibited lower film solubility, especially when the concentration of CLC was higher than 0.25% incorporation (p<0.05). Solubility (water and protein) of the incorporated films decreased as the concentration of CLC increased from 0.25 to 0.5% (p<0.05). However, no significant difference was noted in the incorporated films when the CLC incorporation increased from 0 to 0.25% (p>0.05). The lower film and protein solubility observed in the film suggested that the polypeptides in gelatin underwent more aggregation, leading to more cross-linking. In addition, the establishment of protein and CLC interactions may strengthen the interactions that stabilize the protein net. In general, the water solubility of gelatin film was very high (>90%) when compared with others [9,23,25]. When a film is placed over the food surface, its solubility largely determines the release of antimicrobial compounds [8]. However, incorporating some other component based films into the film forming solution may change the chemical structure of the film, resulting in changes in film and protein solubility. The protein content in film forming solution also affects the film solubility as mentioned by Nur Hanani et al. [25]. They reported that increasing gelatin concentration from 4 to 6% significantly increased the solubility of the film (~40%). Ahmad et al. [9] reported that the incorporation of essential oil might be associated with the hydrophobic nature of oil to interact with the hydrophobic domain of gelatin, leading to increase hydrophobicity of the resulting film. As a result, the solubility of film decreased. Films with high solubility have the potential for developing edible packaging materials intended for easy solubility and release of the incorporated active compounds that exist in the film. However, potential applications may require water insolubility to enhance product integrity and water resistance [26].

**Table 5:** Film solubility and protein solubility of fish gelatin based films incorporated with a combination of catechin and lysozyme at different concentrations

**Treatment (% CLC)**	**Film solubility (%)**	**Protein solubility (%)**
Control	57.51 ± 2.04 ^b^	54.78 ± 13.49 ^b^
0.125 %	56.96 ± 2.69 ^b^	46.94 ± 1.22 ^b^
0.250 %	54.12 ± 1.87 ^b^	29.43 ± 0.87 ^a^
0.500 %	43.96 ± 2.57 ^a^	16.98 ±1.49 ^a^

Values with different superscripts in each column are significantly different (p<0.05).

#### Protein pattern

**Figure 3:**
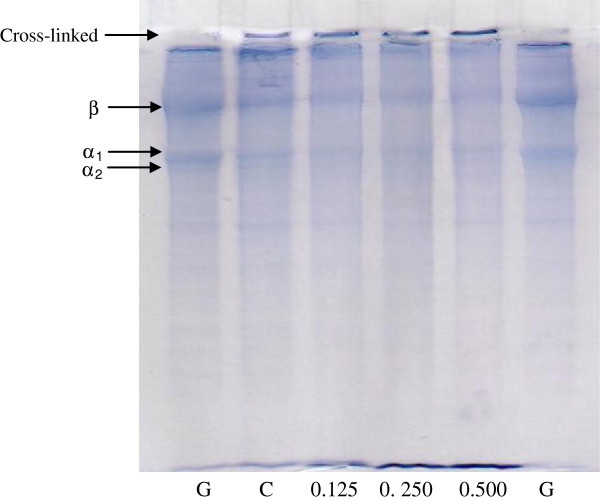
**Protein pattern of fish gelatin based film incorporated with a combination of catechin and lysozyme at different concentrations.** G: Gelatin; C: Control film; Numbers (0.125-0.500) denote the concentrations of the catechin and lysozyme combinations (%).

Protein patterns of gelatin based film incorporated with various concentrations of CLC are shown in Figure 3. High molecular weight (MW) components, including β, α_1_-, and α_2_-components as well as cross-linked constituents were observed in the SDS-PAGE. The starting gelatin (lane G) showed all of the main protein components that are normally found in gelatin samples. When the film was prepared, a slight decrease in band intensity of the β, α_1_-, and α_2_-components were clearly found (lane C). Moreover, cross-linked protein was also observed at the top of the separating gel. Slightly decreased band intensity of β, α_1_- and α_2_-components in the gelatin films was observed when the concentration of CLC increased (lane 0.125-0.5). This was coincidental with the increased protein cross-linking over the separating gel. From this result, CLC might induce the formation of cross-links via covalent bonds. Also, it was suggested that β-chain was more susceptible to cross-linking induced by lysozyme and/or catechin. The cross-linking in the film matrix was more pronounced with increasing CLC concentration. This was in accordance with the continuous decrease in the film’s TS and EAB (Table 1).

### Effect of CLC on film antimicrobial properties

**Figure 4:**
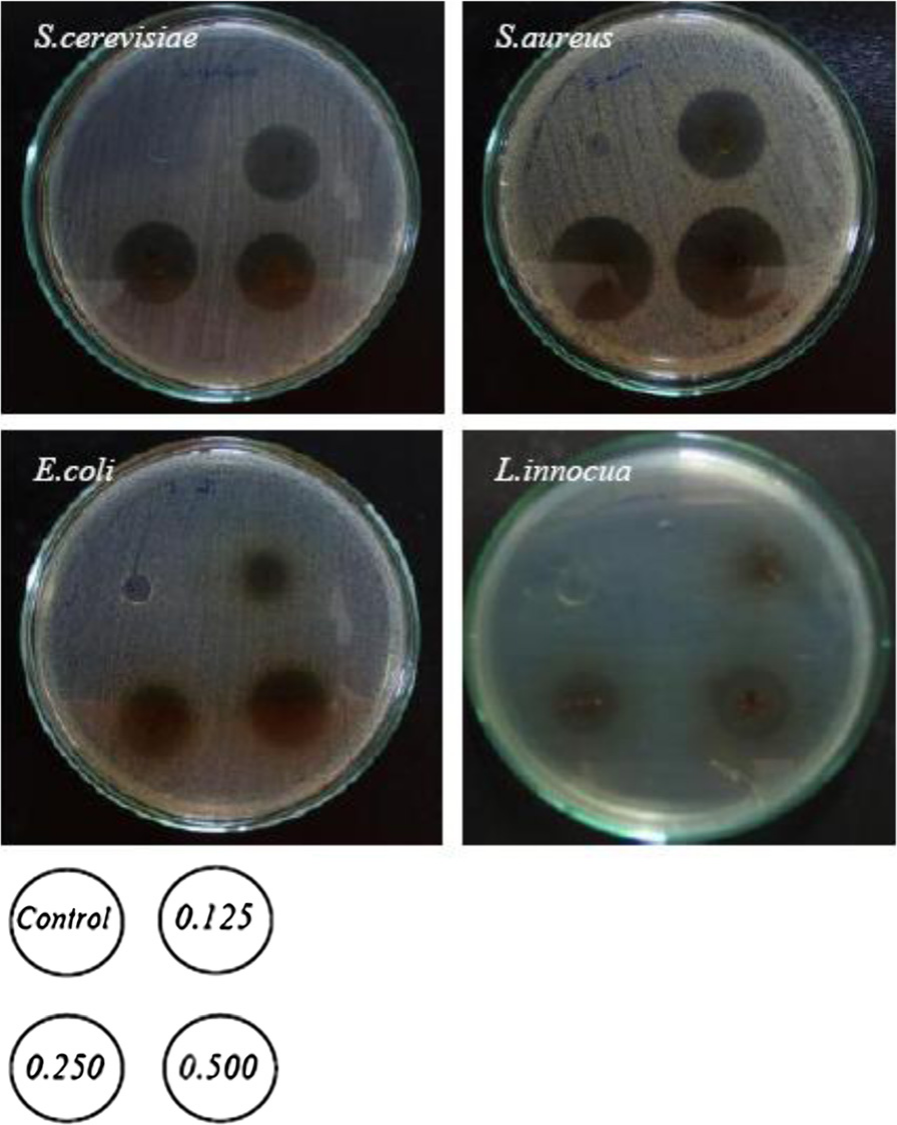
**Antimicrobial activity of fish gelatin based films incorporated with a combination of catechin and lysozyme at different concentrations against *S***. ***cerevisiae***, ***S***. ***aureus***, ***E***. ***coli***, ***and L***. ***innocua.***

Antimicrobial properties of gelatin films incorporated with CLC at various concentrations are presented in Figure 4. CLC showed an inhibition towards all types of tested microorganism: *S*. *cerevisiae*, *S*. *aureus*, *E*. *coli* and *L*. *innocua*. For each microorganism tested, inhibition zones increased with increasing CLC concentration (0.125 to 0.5%), while no antimicrobial activity was found in the control film (without CLC). When comparing all of the microorganisms, *S*. *aureus* was more susceptible to be inhibited by the CLC incorporated film, followed by *S*. *cerevisiae*, *L*. *innocua* and *E*. *coli*, respectively. The higher inhibition for CLC incorporated films was observed for the Gram-positive bacteria (*S*. *aureus* and *L*. *innocua*) and yeast (*S*. *cerevisiae*), while the lower inhibition was found for the Gram-negative bacteria (*E*. *coli*). Lysozyme is an antimicrobial peptide that is effective at preventing Gram-positive (and sometimes Gram-negative) bacteria. Its antimicrobial properties are associated with the hydrolysis of peptidoglycan layers in the bacterial cell wall and also with membrane perturbation [15,27]. Coma [14] reported that lysozyme exhibits antimicrobial activity by splitting the bonds between the N-acetylmuramic acid and N-acetylglucosamine of the peptidoglycan in the bacteria cell wall. Barbiroli et al. [13] also concluded that lysozyme was the most effective at preventing the growth of *E*. *coli*, *L*. *innocua* and *Micrococcus lysodeikticus*. A unique functional barrier to increase the shelf life of food products was discovered from gelatin based film formulated with lysozyme [7].

Green tea polyphenols, especially catechin, have been reported to show inhibitory effects *in vitro* against food spoilage and pathogenic microorganisms including *L*. *monocytogenes*, *E*. *coli*, *Salmonella typhi murium*, *S*. *aureus*, *Shigella frexneri*, and *V*. *Cholera*[28,29]. Catechin has been employed as a food additive for the purpose of antimicrobials and antioxidants for preserving of a wide range of foods, especially meat and meat products. The antimicrobial activity of catechin or any other tea polyphenols is probably due to the inhibition of DNA and RNA synthesis of bacterial cells or may be because of the inhibition of bacteria cytoplasmic membrane function and/or interference with bacteria energy metabolism [12]. In addition, its antimicrobial activity is related to the attack on the phospholipid present in cell membranes, which causes increased permeability and leakage of cytoplasm. It may also be related to their interaction with enzymes located on the cell wall [30].

## Conclusions

The findings suggest that incorporating more CLC into the film could lead to the modified fish gelatin based films, giving them some useful properties (i.e. improving mechanical and barrier properties) and allowing them to be used in areas of high-stretching film applications. However, more work is needed before industrial application. In addition, mixing CLC with film forming solution can improve the performance of gelatin films in terms of antimicrobial properties against food spoilage microorganism.

## Methods

### Chemicals and microbials

Catechin hydrolysate (C1251), lysozyme from chicken egg white (62971), bovine serum albumin (BSA) were purchased from Sigma-Aldrich (St. Louis, MO, USA.). Beta-mercaptoethanol (βME) and electrophoresis reagents were obtained from Bio-Rad Laboratories (Hercules, CA, USA). Glycerol, and other analytical grade reagents were obtained from Merck (Darmstadt, Germany). Mueller-hinton broth (275730) was purchased from Difco (Kansas, USA). Mueller-hinton agar (105437) was purchased from Merck (Darmstadt, Germany).

*Escherichia coli*, *Staphylococcus aureus and Saccharomyces cerevisiae* were obtained from the Biological Laboratory, Scientific and Technological Instruments Center, Mae Fah Luang University, Chiang Rai, Thailand. *Listeria innocua* was purchased from the Department of Medical Sciences, Ministry of Public Health, Bangkok, Thailand. Cultures were streak-plated once a week and a single colony was inoculated into the appropriate media and incubated overnight in the appropriate atmospheric conditions.

### Extraction of fish skin gelatin

Gelatin was extracted from the washed giant catfish skin by the method described in Jongjareonrak et al. [24]. To remove non-collagenous proteins and pigments, the washed skin was soaked in 0.2 mol/l of NaOH with a skin to solution ratio of 1:10 (w/v) at 4°C with continuous gentle stirring. The solution was changed 3 times every 30 min. The alkaline-treated skin was then washed with tap water until it was neutral or slightly basic (pH<7.5). To swell the collagenous material in the fish skin matrix, the alkaline-treated skin was soaked in 0.05 mol/l of acetic acid with a skin to solution ratio of 1:10 (w/v) for 3 h at room temperature (25°C) with continuous gentle stirring. Acid-treated skin was washed as previously described. The swollen fish skin was soaked in distilled water with a skin/water ratio of 1:10 (w/v) at 45°C for 12 h with continuous stirring to extract the gelatin. The mixture was then filtered using two layers of cheesecloth. The resultant filtrate was then freeze-dried. The dried matter that came from the freeze-dried process was ground and then referred to as “gelatin powder”.

### Preparation of gelatin films

The gelatin powder was mixed with distilled water to obtain the film-forming solution (FFS) with a protein concentration of 3% (w/v). Glycerol was used as a plasticizer at a concentration of 25% of protein. The solution was incubated at 60°C for 30 min in a water bath with occasional stirring. After the FFS was cooled to room temperature, the CLC (catechin and lysozyme combination with ratio 1:1) of the FFS at the levels of 0, 0.125%, 0.25, and 0.5% (w/v) were added into the FFS. De-aerated film forming solution (4 g) was cast onto a rimmed silicone resin plate (50x50 mm) and then evaporated at room temperature for 24 h before dried with a ventilated oven environmental chamber (model H110K-30DM; Seiwa Riko Co., Tokyo, Japan) at 25±0.5°C and 50±5% relative humidity (RH) for another 24 h. The obtained dried films were manually peeled.

### Analyses of gelatin film properties

#### Film thickness

The film thickness was measured with a hand-held micrometer (Bial Pipe Gauge, Peacock Co., Tokyo, Japan). Nine random locations around each of the ten film samples were used for thickness determination.

#### Mechanical properties

Prior to testing the mechanical properties, the films were conditioned for 48 h at 50±5% RH at 25°C. The tensile strength (TS) and elongation at break (EAB) were determined by using a Universal Testing Machine (Lloyd Instrument, Hampshire, UK). Ten samples (2x5 cm) with an initial grip length of 3 cm were used for testing. The cross-head speed was set at 30 mm/min with 100N load cell use.

#### Water vapor permeability

The films’ water vapor permeability (WVP) was measured by using a modified ASTM [31] as described by Shiku et al. [32]. The films were sealed onto a permeation cup containing silica gel (0% RH) with silicone vacuum grease and an O-ring to hold the film in place. The cups were then placed in a desiccator saturated with water vapor at 30°C. The cups were weighed at 1 h intervals over a period of 8 h, and the films’ WVP was calculated as follows [33]:

(1) $$\text{WVP}=wx{\text{A}}^{-1}{\text{t}}^{-1}{{(\text{P}}_{2}{-\text{P}}_{1})}^{-1}$$

w is the weight gain of the cup (g); x is the film thickness (m); A is the area of exposed film (m^2^); t is the time of gain (s); and (P_2_-P_1_)^-1^ is the vapor pressure differential across the film (Pa). The WVP was expressed as g m^-1^ s^-1^ Pa^-1^. A total of five samples were determined for each film.

#### Colour, light transmission and film transparency

The colour of the film was determined with a Hunter lab colour meter (Colour QuestXE, Virginia, USA) and expressed as L* (lightness), a* (redness/greenness), and b* (yellowness/blueness).

The ultraviolet and visible light barrier properties of the films were measured at selected wavelengths between 200 and 800 nm by using a UV-16001 spectrophotometer (Shimadzu, Kyoto, Japan) according to the method described by Jongjareonrak et al. [24]. The film transparency was calculated by the following equation [34]:

(2) transparency=−log T600/x

T600 is the fractional transmittance at 600 nm, and x is the film thickness (mm).

#### Surface morphology and gelatin film microstructure

The surface morphology of the gelatin films was examined by using a Canon Powershot A95 digital camera at 5.0 megapixels and illuminant D65 (Canon Marketing (Thailand) Co.,Ltd., Bangkok, Thailand). The gelatin films’ microstructure was observed by using a scanning electron microscope (SEM) (LE01450VP, Cambridge, UK) at an acceleration voltage of 10 kV and magnification at 1,200x and 5,000x.

#### Film solubility and protein solubility

The film solubility was determined according to the method of Gennadios et al. [35]. The conditioned film samples were weighed and placed in a 50 ml centrifuge tube containing 10 ml of distilled water. The mixture was shaken at a speed of 250 rpm using a shaker (Heidolth Inkubator 10000, Schwabach, Germany) for 24 h. The un-dissolved debris was then removed by centrifugation at 3,000g for 20 min. The pellet was dried at 105°C for 24 h and weighed. The weight of the solubilized dry matter was calculated by subtracting its difference from the initial weight of the dry matter. It was then expressed as a percentage of the total weight.

To determine the protein solubility, the protein concentration in the supernatant after centrifugation was determined by using the Biuret method [36]. Protein solubility was expressed as the percentage of the protein content of the film dissolved in water to the total protein in the film, which was solubilized with 1 M of NaOH for 24 h.

#### Electrophoretic analysis

The protein patterns of gelatin, film forming solution, and the gelatin films were all analyzed by SDS–PAGE according to the method of Laemmli [37]. To solubilize the films, the samples were mixed with a solubilizing solution (1% SDS). The mixtures were heated at 85°C for 1 h in a water bath to dissolve the proteins. The solubilized samples were then mixed with a sample buffer (0.5 M Tris–HCl, pH 6.8 containing 4% (w/v) SDS, and 20% (v/v) glycerol) and 10% (v/v) βME at a ratio of 1:1 (v/v). The samples (16 μg protein) were loaded into the polyacrylamide gel made of 7.5% running gel and of 4% stacking gel and subjected to electrophoresis at a constant current of 15 mA per gel by using a Mini Protean II unit (Bio-Rad Laboratories, Inc., Richmond, CA, USA). After electrophoresis, the gel was stained with 0.05% (w/v) Coomassie blue R-250 in 15% (v/v) methanol and 5% (v/v) acetic acid and then destained with 30% (v/v) methanol and 10% (v/v) acetic acid.

#### Antimicrobial testing of gelatin films

The films were tested for their inhibition against the target microorganisms: *L*. *innocua*, *S*. *aureus*, *E*. *coli* and *S*. *cerevisiae* by using an agar disc diffusion method. Cell densities of 10^8^ cfu mL^−1^ were calculated and prepared from the cultures at approximately 7.50×10^8^ cfu mL^−1^ for *L*. *innocua* and *E*. *coli*; 4.85×10^8^ cfu mL^−1^ for *S*. *aureus*; and 4.75×10^8^ cfu mL^−1^ for *S*. *cerevisiae*. Each film sample was cut into a circle of 5 mm in diameter and sterilized with UV light for 30 min [38] prior to being placed on an agar plate surface seeded with the test cultures. The plates were incubated for 1–2 days at the appropriate temperature for each culture. A clear zone formed around the film disc in the medium, and it was recorded as an indicator of inhibition for the microbial species. The evaluation of the inhibitory activity was carried out in quadruplicate by observing the inhibition zones.

### Statistical analysis

The data was subjected to analysis of variance (ANOVA). A means comparison was carried out by Duncan’s multiple range tests. The analysis was performed by using an SPSS package (SPSS 10.0 for window, SPSS Inc, Chicago, IL).

## Competing interests

There are non-financial competing interests (political, personal, religious, ideological, academic, intellectual, commercial or any other) in relation to this manuscript to declare.

## Authors’ contributions

SR carried out the whole work experimental design, chemical testing of the film, analysis and interpretation of data and manuscript drafting and revising. PS carried out the design and testing of the film in the anti-microbial parts, analysis and interpretation of data. DK carried out the design and physical testing of the film in the experiments, analysis and interpretation of data. SB participated in giving advises and final approval of the manuscript. All authors read and approved the final manuscript.
